# Health technology assessment in middle-income countries: recommendations for a balanced assessment system

**DOI:** 10.3402/jmahp.v2.23181

**Published:** 2014-03-11

**Authors:** Dávid Dankó

**Affiliations:** Corvinus University of Budapest, Institute of Management, Budapest, Hungary

**Keywords:** health technology assessment (HTA), middle-income countries (MIC), balanced assessment, multi-criteria decision analysis (MCDA), pharmaceutical assessment, reimbursement decisions

## Abstract

Because of significant differences in institutional contexts, health technology assessment (HTA) systems that are in place in core pharmaceutical markets may not be suitable, fully or in part, for middle-income countries (MICs) and for other noncore markets. Particular challenges may arise when systems based on the economic evaluation paradigm are conceptualized and implemented in MICs, sometimes with an insufficient level of awareness of the local institutional factors that influence pricing and reimbursement decision making. Focusing on pharmaceuticals, this article investigates possible development directions for HTA systems in MICs and noncore markets bearing similar institutional characteristics, and it provides recommendations for a balanced assessment system (BAS). For this, the main paradigms of HTA have also been reviewed briefly and factors influencing HTA and pricing and reimbursement decisions in MICs and in similar noncore countries have been summarized. The proposed BAS framework takes into account available resources and capabilities and is supposed to facilitate access to new pharmaceuticals while ensuring the transparency of decision-making processes and the stability of the pharmaceutical budget.

Broadly defined (1), health technology assessment (HTA) is the ‘systematic evaluation of medical technologies regarding their effectiveness, appropriateness, efficiency as well as social and ethical aspects and implications.’ In a similar view (2), HTA is ‘the systematic evaluation of properties, effects, and/or impacts of health-care technology. It may address the direct, intended consequences of technologies as well as their indirect, unintended consequences. Its main purpose is to inform technology-related policymaking in health care. HTA is conducted by interdisciplinary groups using explicit analytical frameworks drawing from a variety of methods.’ Thus, HTA has a decision-support role in pricing and reimbursement decision making^1^ by providing responsible bodies and organizations with timely, accurate, and sound information on new medical technologies (3, 4). Besides this, HTA can also be linked to the management of the reimbursement list (i.e., subsequent reimbursement reviews).

HTA covers pharmaceuticals, medical devices, as well as all clinical procedures, including surgical interventions and diagnostics. In most countries, however, HTA focuses on pharmaceuticals and medical devices because these are standardized technologies whose ownership is clear; they pose a high financial burden on health-care systems, and their social impact and visibility are both significant. Within the pharmaceutical domain, the key questions to which HTA should provide an answer are: ‘Is it worth spending public money on a medicine? If yes, to what extent, and for which patients?’.

Over the years, three main paradigms of HTA have evolved across developed health-care markets (5–7):

1. *Economic evaluation* is the dominant paradigm in countries in which legislation or assessment practices have endorsed the mainstream pharmacoeconomic (PE) tradition. Cost-effectiveness and, lately, budget impact are the focus of pharmaceutical assessment, which itself is quantitative and heavily relies on biostatistics and PE modeling (8). Methodological rigor and assumed objectivity are often mentioned as strengths of this paradigm, although possible drawbacks or shortcomings can be financial bias, ‘methodology-heaviness’, low understandability for higher-level decision makers, lack of sufficiently robust criteria for determining cost-effectiveness, constraints in the international transferability of results, and resource-intensiveness (9, 10). Economic evaluation can exist in a ‘heavy’ form in which a state agency performs single technology assessments (STA) as comprehensive multiple technology assessments (MTA), and in a ‘light’ form, where assessments are only triggered by submissions by pharmaceutical companies^2^ (STA only) (11). The reference institution for the economic evaluation approach has traditionally been the UK's National Institute of Care and Health Excellence (NICE, heavy model).
2. *Qualitative/comparative assessment* bears more resemblance in its approaches to the regulatory logic. Clinical aspects focus on pharmaceutical assessment, but other criteria such as societal impact can also be considered. Strengths are a heavy emphasis on therapeutic value added (as opposed to cost-effectiveness), avoidance of methodology-heavy decision making in favor of collective expert judgment, better understandability for higher-level decision makers (compared to economic evaluation), and easier customization of decision criteria to local characteristics. Possible shortcomings are low transparency, inconsistent methodologies (e.g., scoring systems with irrelevant criteria), less rigorous decision-making processes, and the exclusion of economic aspects in decision making.
3. *Balanced assessment* sets out to integrate aspects from economic evaluation and from qualitative/comparative assessment on the basis of multicriteria decision making (12), striving to eliminate methodological biases associated with the two ‘stand-alone’ paradigms. In general, balanced assessment systems (BASs) tend to take into account economic factors (cost-effectiveness, budget impact), therapeutic value added, societal impacts and alignment with health policy priorities. Economic metrics are used as inputs into multicriteria decision analysis (MCDA); collective decision making, traceability, and process transparency are encouraged. Balanced assessment can also be established in ‘heavy’ and ‘light’ models based on primary analyses; in these cases, resource need is going to be high or very high. Alternatively, it can follow an ‘ultralight’ model, whereby expert checklists and secondary assessments are widely used, with assessors reaching back to prior international assessments (6). The submission of economic dossiers is not always required although it is always permitted. Figure 1 gives an overview of the three major HTA paradigms.

**Fig. 1:**
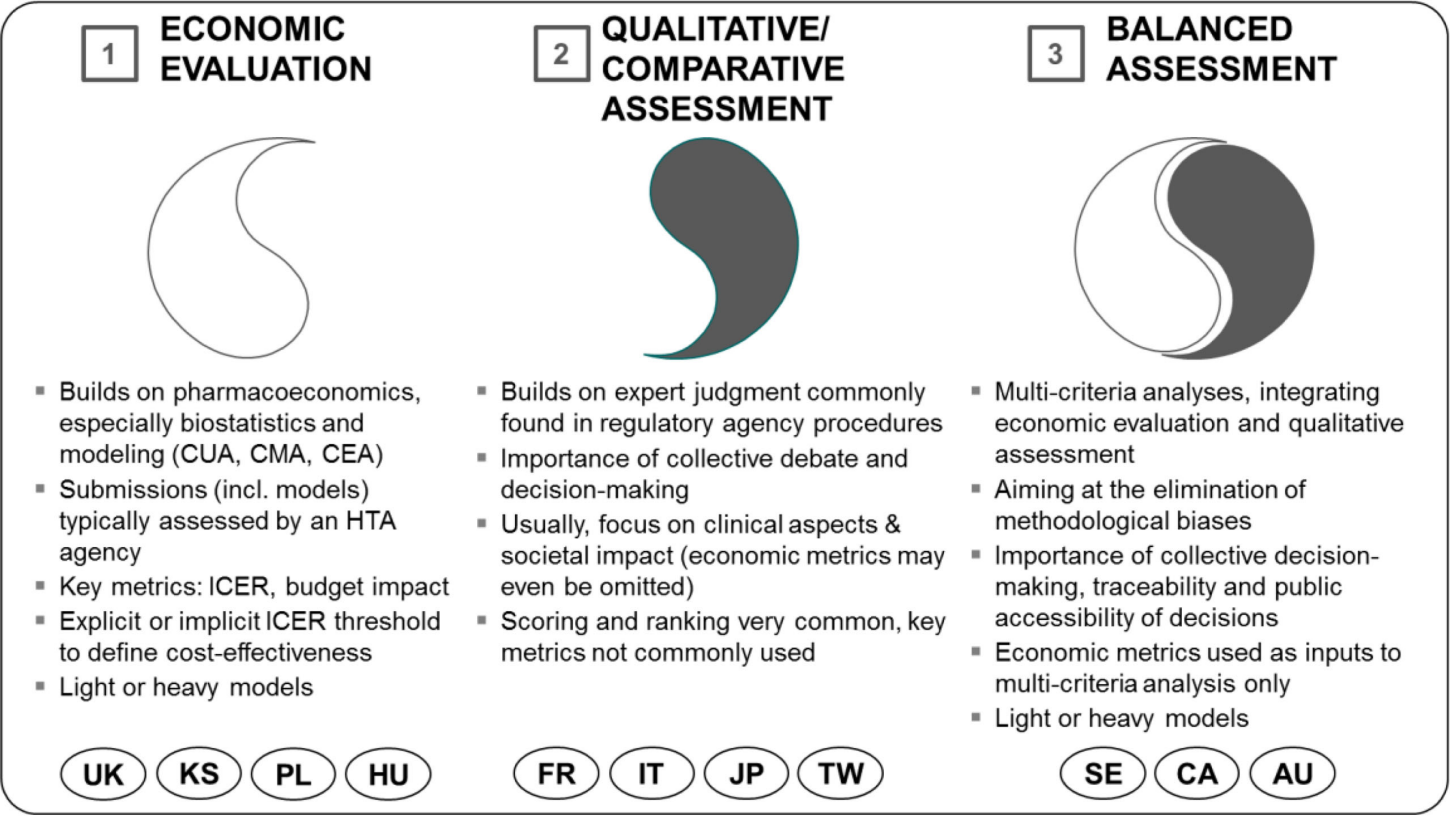
Three paradigms of health technology assessment (5). UK, United Kingdom; PL, Poland; HU, Hungary; KS, South Korea; FR, France; IT, Italy; JP, Japan; TW, Taiwan; SE, Sweden; CA, Canada; AU, Australia. Note: Countries are not clear ‘archetypes’ themselves; instead they are shown at the paradigm to which they are closest (13).

## Institutional context in MICs and other noncore pharmaceutical markets

Middle-income countries (MICs) are commonly defined following the World Bank's income group approach which classifies countries based on gross national income (GNI) per capita.^3^ Lower MICs have GNI per capita values between US$1.036 and 4.085, while the same indicator for upper MICs is between $4.086 and 12.615. MICs are not a homogeneous cluster—there are hugely different countries and markets that fall under this term. For example, Turkey, China, Brazil, Hungary, Thailand, Malaysia, South Africa, Ukraine, Morocco, Mexico, Gabon, Nigeria, and Colombia are all MICs. However, MICs have several social, technological, and development characteristics (14) that result in similar institutional setups in pharmaceutical policy-making and implementation across the very different countries.

The picture is further complicated by the fact that some nominally high-income markets (e.g., Central Europe, Russia, Gulf countries, Chile, Uruguay) still show distinct ‘middle-income characteristics’ or are in a transition phase toward social, technological, and development patterns peculiar to established high-income markets. There is no appropriate collective term to denote these countries. Sometimes they are called emerging markets (which can be considered a bit anachronistic or Eurocentric) or, alternatively, ‘non-core pharmaceutical markets’ is also a possible term reflecting their share in global pharmaceutical sales. For purely practical reasons, we will use the term MICs to refer to the MIC income group and other noncore pharmaceutical markets.

Some MICs already have fully or partially institutionalized HTA systems in place (e.g., Poland, Hungary, Thailand, Brazil), others apply PE criteria in decision making but not in a structured form (e.g., Serbia, Czech Republic, Bulgaria, Turkey), and others do not have HTA systems yet (e.g., South Africa, Ukraine, Gulf countries) (15, 16). Most MICs seem to have chosen their peer countries based on global academic representation (e.g., UK, Canada, Australia—not independently from the influence of English-language HTA literature), cultural linkages, and know-how transfers via international development initiatives (e.g., France, Sweden or, to a lesser extent, the Netherlands) (6). However, the HTA paradigms established in these countries may not function properly in MICs or may not be suitable for know-how transfer because of the different institutional backgrounds (17). We see that major differences are related to politicized decision making and the low acceptance of methodology heavy HTA, the quality of relevant local data, the scarcity of experts and capabilities and, as a result of these, to low structure in pricing and reimbursement decision making (18).

- *Politicized decision making*. In a ‘typical’ MIC, there are few independent, specialized, decision-making bodies. Instead, there are many payers at several different levels, most of whom lack the HTA background that would give them a chance to interpret complex decision-support analyses. Some of them regard methodology heavy, ‘black box’ HTA approaches with outright suspicion. Within such systems, budget pressure and the degree of politicization in reimbursement policy shift actual decisions up to ministerial (or sometimes higher) levels, where *analytical* approaches tend to be irrelevant for decision makers who are mostly *intuitive*. In many instances, severe budget pressures lead the few analytical decision makers to focus on budget impact exclusively, which introduces a dysfunctional bias into how HTA, and economic evaluation in particular, is applied and interpreted (19).
- *Local data availability*. Local epidemiology data is often missing or incomprehensive, or it may be inconsistent or significantly distorted by diagnosis-related groups (DRG)-coding bias (20). Partially available data means that for certain therapeutic areas epidemiology data is not collected; DRG-coding bias means that hospitals and outpatient centers chronically overreport patient numbers for complicated cases (for which DRG payments are higher). In addition, patient pathways are often undefined, which makes it very complicated to assess the performance of health-care subsystems. Limited availability of reliable local data and undefined patient pathways also mean that international PE models prepared for new pharmaceutical products are difficult to adapt in a way that they could effectively support reimbursement decision making.
- *Lack of experts and capabilities*. The number of trained HTA experts is relatively limited in larger MICs and very low in most of the smaller MICs.^4^ Normally, basic PE education is available for pharmacy students at the main medical universities. A small number of HTA experts work as consultants for the authorities and with pharmaceutical companies.
- *Low structure*. As a result of the previous three factors, few explicit frameworks and guidelines are used in many MICs during pricing and reimbursement processes (21). Decisions are taken based on (often ad hoc) expert opinion and regulatory requirements. In many countries, key requirements for cost-effectiveness analysis, budget impact calculations, or MCDA have not been clearly defined yet. Consequently, the PE data submitted by pharmaceutical companies varies considerably, and the robustness of the subsequent assessment is contingent upon the quality of the dossier.

For MICs, these challenges have important consequences:

- Economic evaluation is often misaligned with the needs and possibilities of MICs, and it can become a significant barrier in patient access to new pharmaceuticals. The biggest risk associated with economic evaluation is that, because of distorted cost relationships within the health-care system (in particular, outdated technologies serving as ‘standard practice’ comparators and low wages of health-care professionals), most value-added pharmaceuticals will *not* end up being cost-effective in MIC, and budget-constrained decision makers may use this as a basis to deny access to new treatments across the several stages of the pricing and reimbursement process. This inherent problem cannot effectively be tackled through adjustments to the acceptable incremental cost-effectiveness ratio (ICER) threshold value or range; however, such methodological efforts may indeed cause undesirable pricing incentives (22). Second, there is a high risk that economic evaluation will increasingly focus on pharma budget impact and will consequently contribute to a reinforcement of the fiscal mind-set and silo thinking (23) in reimbursement decision making. Third, some methodological dilemmas inherent in economic evaluation (9) (e.g., comparator choice, health benefit assessment, sensitivity to statistical procedures used) and transferability constraints of economic evaluations (24–26) may result in the delegitimation of HTA with higher level payers. As a side effect, questions and concerns related to the misinterpretation (or faulty interpretation) of the economic evaluation conclusions may consume a considerable amount of time, further postponing access to new pharmaceuticals.
- In the context of severe budget constraints, qualitative/comparative assessment tends to be insufficient because it underemphasizes financial aspects. Most of the world's qualitative/comparative assessment systems are geared toward assessing clinical value and improvement offered by the therapy. In an MIC, where budget restrictions may be the most important driver behind reimbursement decisions, financial aspects can hardly be disregarded. This speaks for a balanced (multicriteria) combination of financial and nonfinancial aspects in decision making.
- Models based on extensive primary data gathering and analysis have a limited chance of being successful because of the lack of necessary resources and competences. In MICs, there are not enough experts and systems to perform such resource-intensive analyses. Moreover, in some countries, there may be a high fluctuation in government bodies because HTA experts are employed as public servants, typically with salaries that are not competitive with remuneration levels in for-profit companies.

These characteristics lead to two important conclusions impacting paradigm choice in MICs. First, conceptual and methodological deficiencies make it unlikely that either economic evaluation or qualitative/comparative assessment *alone* would be sufficient and/or able to support reimbursement policy effectively in MICs. Second, limited HTA resources and capabilities tend to rule out ‘heavy’ and, depending on development level, even ‘light’ models that base their recommendations on primary data gathering and analysis. In many instances, these HTA approaches are themselves not ‘cost-effective’ in MICs (i.e., the value of reduction in informational uncertainty that they are able to provide is lower than the cost of implementing and maintaining these systems).

## BASs for MICs: framework and process

Based on the aforementioned considerations, MICs may need to follow their own approaches or at least take utmost care in adopting pharmaceutical assessment models from core pharmaceutical markets. It might be appropriate for MICs to introduce a system that uses (1) a sustainable combination of financial and nonfinancial decision criteria, (2) in a resource-conscious and technically implementable way, in line with local resources and capabilities.

Next, we will outline a possible conceptual framework for such a BAS in MICs, noting that this approach is under no circumstance the only best way to follow.^5^ Depending on local priorities, there can be several possible model implementations for balanced systems, providing that the underlying design principles are followed. It is also worth noting that pharmaceutical assessment is generally not necessary for bioequivalent generic products. For nonbioequivalent generics and biosimilars, some countries may consider simplified assessment as a prerequisite for reimbursement, while others may omit assessment entirely.

**Fig. 2:**
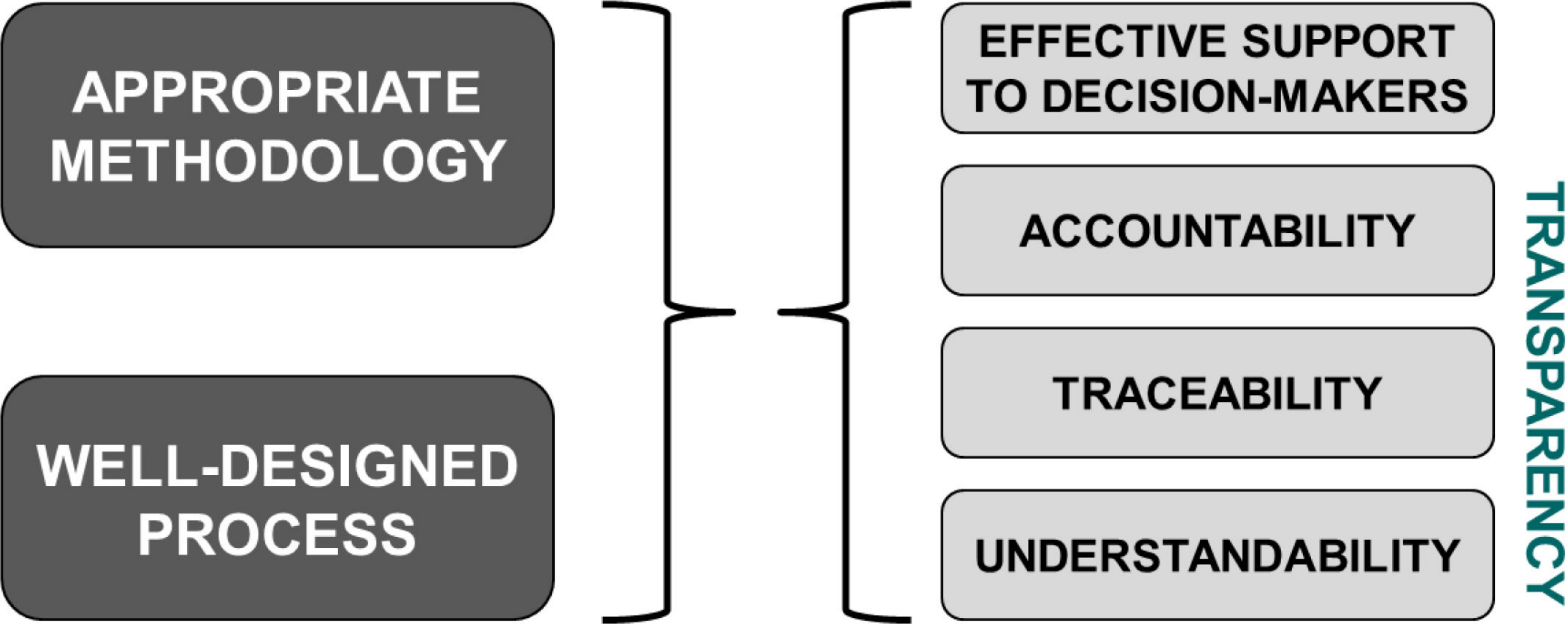
Transparency is dependent on appropriate methodology and a well-designed process.

A BAS requires a proper combination of two things: an easy-to-use methodology and a well-designed assessment process (Fig. 2):

- *Methodology* must be selected in a way that (1) persons who are responsible for assessment can use it without specialized education; (2) higher level decision makers are also able to understand and follow it. This means that secondary analyses will be very important—relevant prior decisions should be researched, aggregated, and synthesized. Primary analysis based on economic models is only encouraged in cases when detailed local economic evidence is indispensable for decision making, or there are no available secondary analyses. This approach strives for a balance among methodological convenience, local capabilities, and alignment with decision-making styles of higher level decision makers.
- *Process*. The assessment process must be designed in a way that participating organizations are accountable for their decisions; these decisions are traceable and underlying considerations are understandable for all stakeholders. This will also require a ‘linearization’ of the pricing and reimbursement process.

**Fig. 3:**
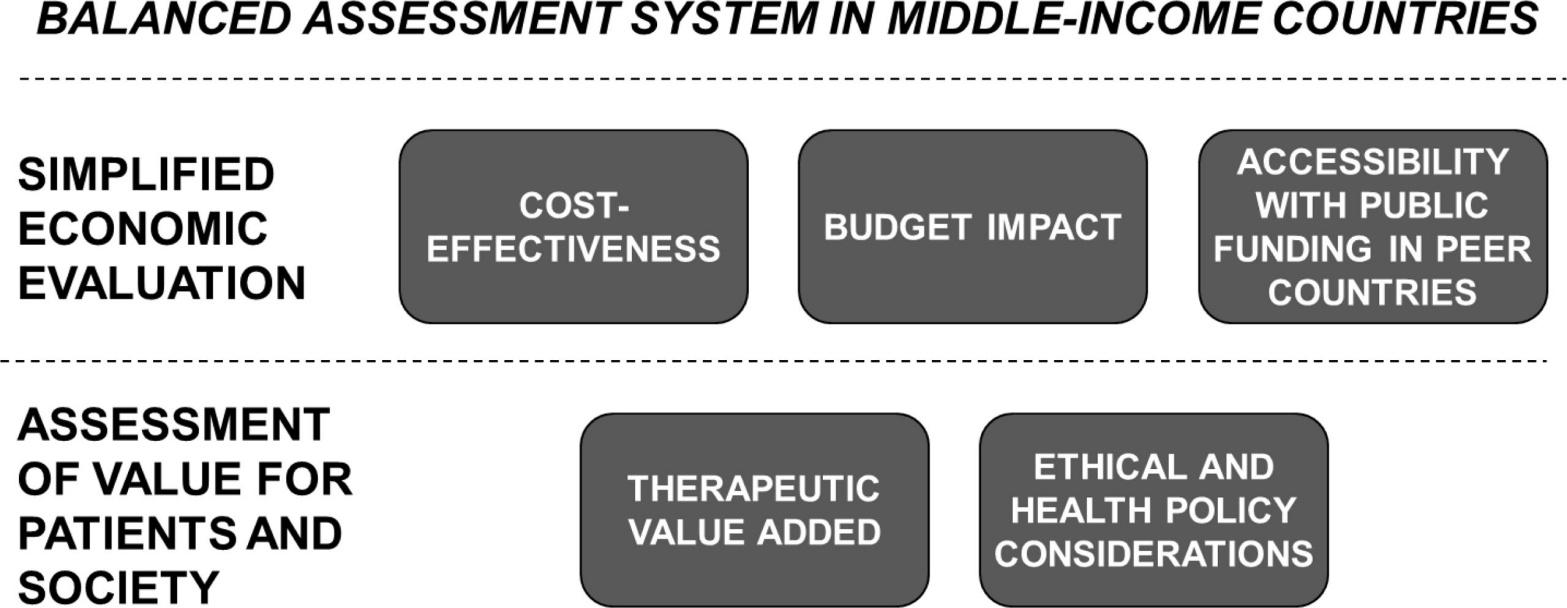
Balanced assessment system (BAS) in middle-income countries (MICs).

Regarding *methodology*, MICs may consider translating the principles of a BAS into a *multicriteria assessment grid*. An example of such an assessment grid is shown in Table 1. The grid is essentially a catalogue of quantitative and qualitative aspects grouped in such a way as to reflect five relevant drivers behind a pricing and reimbursement decision: (1) cost-effectiveness, (2) therapeutic (clinical) value added, (3) ethical and health policy considerations, (4) accessibility with public funding in peer countries, and (5) budget impact. Cost-effectiveness, budget impact, and accessibility in peer countries can be bundled into *simplified economic evaluation*, while therapeutic value added together with ethical and health policy considerations can be covered by an *assessment of value for patients and society* (Fig. 3).

**Table 1:** Example of a BAS assessment grid for a hypothetical middle-income EU member state

Dimension	Example criteria	Score
	Simplified economic evaluation Within each dimension, only one statement can be valid for the assessed pharmaceutical	
A. Indicators of cost-effectiveness	1. The pharmaceutical has been assessed to be cost-effective in the indication submitted for public funding in a payer-commissioned economic evaluation by a leading HTA agency	TBD
	2. The pharmaceutical has been assessed to be cost-effective *in part of* the indication submitted for public funding in a payer-commissioned economic evaluation by a leading HTA agency	TBD (but lower than 1)
	3. All other cases	0
B. Budget impact	4. Primary budget impact analysis substantiates with reasonable robustness that the pharmaceutical will save resources in public pharmaceutical expenditure	TBD
	5. Primary budget impact analysis substantiates with reasonable robustness that the pharmaceutical will save resources in the total health care	TBD (but lower than 4)
	6. Primary budget impact analysis substantiates with reasonable robustness that the pharmaceutical will not increase public pharmaceutical expenditure by more than ‘X’ (or ‘Y%’)	TBD (but lower than 5)
	7. All other cases	0
C. Accessibility with public funding in peer countries	8. The pharmaceutical is accessible with public funding in at least ‘X’ peer countries (as defined by national legislation)	TBD
	9. The pharmaceutical is accessible with public funding in less than ‘X’ but more than ‘Y’ peer countries (as defined by national legislation)	TBD (but lower than 8)
	10. All other cases	0
	Assessment of value for patients and society Only statements 11 and 12 and 13 and 14 are mutually exclusive	
D. Therapeutic value added	11. The pharmaceutical has been found to offer important therapeutic/clinical benefit by one or more leading HTA agency/agencies	TBD
	12. The pharmaceutical has been found to offer modest therapeutic/clinical benefit by one or more leading HTA agency/agencies	TBD (but lower than 11)
	13. The side effect profile of the pharmaceutical is substantially more favorable than that of the comparator therapy,* considering frequency, severity, and health burden of side effects	TBD
	14. The side effect profile of the pharmaceutical is somewhat more favorable than that of the comparator therapy,* considering frequency, severity, and health burden of side effects	TBD (but lower than 13)
	15. The pharmaceutical company has substantiated through publicly available data for at least ‘X’ peer countries that the real-life therapeutic effectiveness of the pharmaceutical is superior to the real-life therapeutic effectiveness of the comparator* product	TBD
	16. The pharmaceutical company has substantiated that the medicinal product improves ease-of-use (convenience for patients) in comparison with the comparator* product	TBD
E. Ethical considerations and health policy priorities	17. The reimbursement application is submitted with an indication that it has been declared a primary public health priority by national health-care authorities	TBD
	18. The pharmaceutical holds an orphan designation	TBD
	19. The reimbursement application is submitted in a pediatric indication	TBD
	20. The reimbursement application is submitted in a therapy area where no new active substance has been accepted for public funding in the past ‘X’ years	TBD

* Comparator choice: As a main rule, the comparator should be the most widely used reimbursed medicine in a country for which the product seeking reimbursement offers a therapeutic alternative. If the product seeking reimbursement does not substitute any already reimbursed product, the comparator should be the most widely available standard (palliative, supportive, non-medicinal, etc.) therapy. If no treatment is available, palliative care should be used as a comparator. If the product seeking reimbursement has any alternative(s) from the same ATC4-level group(s) (=analogue) that is (are) already reimbursed in essentially the same indication, then the comparator should be the lowest priced product in this set of alternatives. The lowest price should be calculated as daily cost of therapy on a prescribed daily dose (PDD) basis depending on the SMPC. (BAS, balanced assessment system; HTA, health technology assessment.)

Regarding the assessment grid shown in Table 1, the following comments will be relevant:

- *Cost-effectiveness* can be acknowledged directly (based on a country-specific study) or indirectly (based on prior assessments by international HTA agencies). Despite limitations in the transferability of HTA results, prior assessments in more developed markets can be considered as proxies. Geographical scope should be wide; for example, European MICs can take into account guidance from non-European countries, resources permitting. Based on the considerations noted earlier, pricing and reimbursement bodies should not make the submission of country-specific health economics models a prerequisite for reimbursability.
- *Accessibility with public funding in peer countries* should not be screened across an arbitrarily designed basket of reference countries (as is the current practice in many MICs). Although it might seem simplistic, it is generally less biased, and more indicative, to refer to the *number* of peer countries where the medicine is already publicly funded.
- *Budget impact* should be captured across all subbudgets of the national health-care budget (including hospitals, primary care, sick leaves, etc.); however, preferences may be attached to pharmaceuticals that directly save resources in public pharmaceutical expenditure. If patient numbers, treatment durations, or individual doses carry strong uncertainty, guarantees via managed entry schemes may be required from pharmaceutical companies.
- In a BAS, factors of *therapeutic value added* should be explicitly analyzed and assessed as information aggregated into quality-adjusted life years (QALYs) or similar health economics metrics are usually insufficient for decision makers to understand the true benefits of new pharmaceuticals and to prioritize them. In particular, at least five major components of therapeutic value added can be recognized: the ability of the pharmaceutical product to treat previously incurable conditions, superior clinical outcome (higher efficacy), more favorable side effect profile, evidence of real-life therapeutic effectiveness, and ease-of-use (convenience). These components can of course be weighted in a differentiated way.
- *Ethical and health policy considerations* should be covered through country-specific criteria. Only illustrations are provided in Table 1, and several variations are possible.

In the assessment grid, each aspect (criterion) should be assigned an individual score. If the new pharmaceutical product complies with a criterion, it is supposed to receive the individual score assigned to that criterion. If it does not meet a criterion, it should receive a zero score for that aspect. Individual scores should then be aggregated into a total score, with the reimbursement decision strongly correlating with this total score. An illustrative outcome table (see Table 2) differentiates among unconditional reimbursement, conditional reimbursement, and no reimbursement. Theoretically, maximum allowable prices and/or reimbursement rates may also be linked to the scoring algorithm (as is the case in France's SMR/ASMR system), although price negotiations may be a more realistic option in MICs.

**Table 2:** Example of an outcome table in a BAS

Total score (calculated as the sum of individual scores)	Listing decision
0–49	Not reimbursable
50–69	Conditional reimbursement with programmed reimbursement review within 18–24 months *or* reimbursement with real-life effectiveness guarantee (in a managed entry scheme)
70 and above	Unconditional reimbursement in the indication requested

BAS, balanced assessment system.

The design of the assessment grid in Table 1 is based on the assumption that two large clusters of pharmaceuticals are preferred by public payers: (1) pharmaceuticals with considerable therapeutic value added (usually sold at a premium price) and (2) pharmaceuticals that are noninferior to an already reimbursed comparator but that save resources either in the pharma budget itself or more generally in the health-care budget(s). Medicinal products in both large clusters should receive reasonably close to unconditional reimbursement. Other pharmaceuticals, which do not have such distinctive value proposals, are expected to offer additional benefits along other criteria.

The accepted and implemented version of the assessment grid should be based on the widest available consensus. Although applying the grid must be straightforward in itself, all generalizations and simplifications must be avoided that would lead to unfounded or arbitrary pricing and reimbursement decisions. This also means that the final version of the grid should preferably be elaborated through a series of expert discussions among ministries, health service/insurance authorities, medical and pharmacy unions, trade associations, and patient associations.

**Fig. 4:**
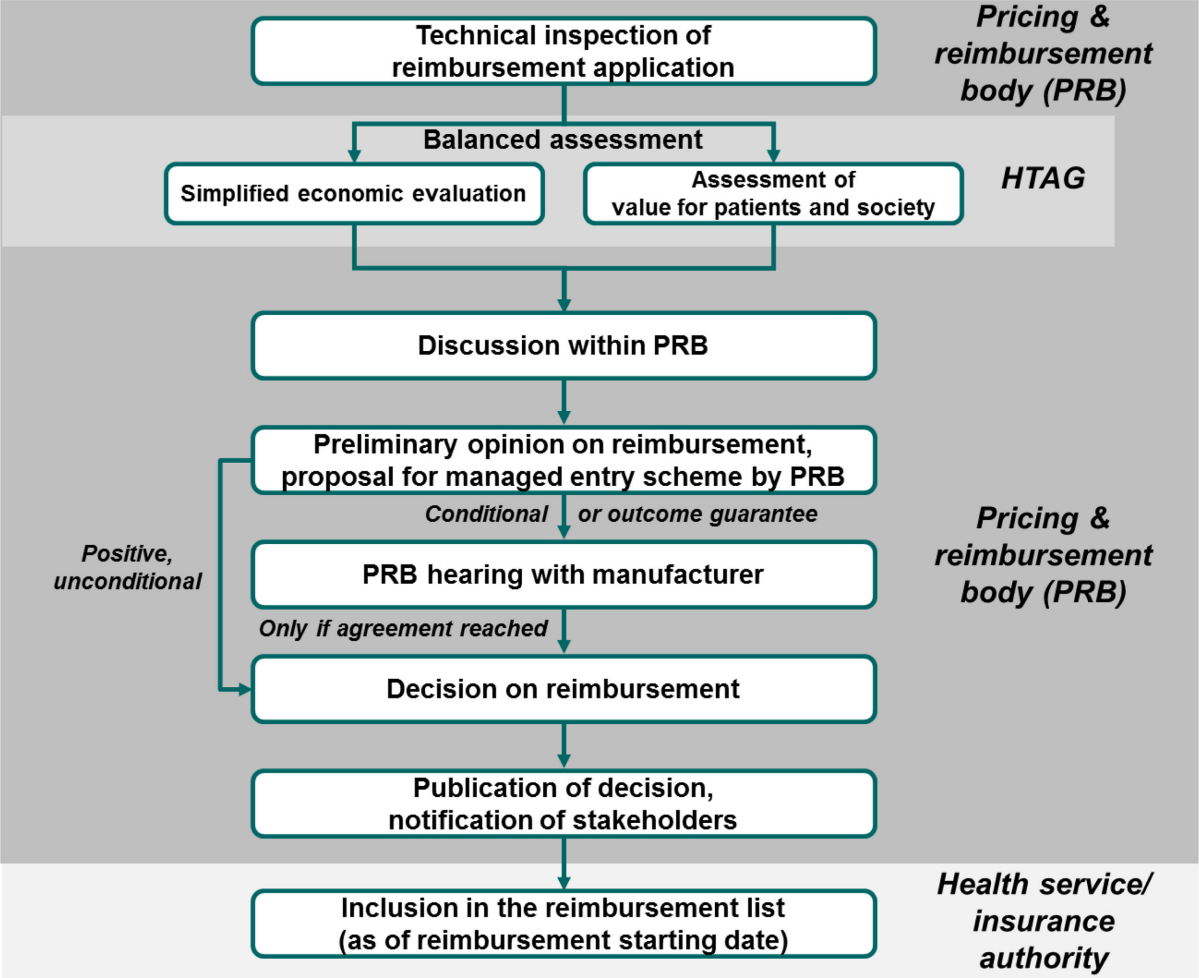
Possible high-level scheme for the pricing and reimbursement process in a middle-income country. PRB, pricing and reimbursement body; HTAG, health technology assessment group. Notes: (1) The process only applies to reimbursement decisions for new pharmaceuticals. Price-only applications may be dealt with in a much simpler system.

Regarding the *process* of assessment and decision making, we propose a linear framework, as shown in Fig. 4, that incorporates various elements from major pharmaceutical markets.^6^ The process starts with a submission (dossier) by the pharmaceutical company to the pricing and reimbursement body (PRB) that contains, at least, the requested list price, the requested reimbursement indication, and all the PE and clinical evidence necessary for BAS assessment. The PRB carries out a technical inspection and, if the dossier is complete, it forwards all documentation to a health technology assessment group (HTAG) that, preferably, operates independently from the PRB.

The main difference between a PRB and an HTAG is that a PRB is a decision-making body that negotiates with pharmaceutical companies and takes pricing and reimbursement decisions, whereas an HTAG is an expert body that carries out a BAS as the most important input to the PRB's subsequent decision. HTAGs are therefore best set up as independent bodies of specialized civil servants (see following) with or without support from academic institutions. HTAG decisions must not be influenced by a PRB, otherwise the PRB's position and eventual budget-related considerations would influence the objectivity of the HTAG assessment. HTAGs themselves can consist of two subgroups, the first one performing simplified economic evaluation (cost-effectiveness, international reimbursement references, budget impact) and the second one assessing value for patients and society.

The HTAG prepares an assessment summary that includes key findings from the simplified economic evaluation and the assessment of value for patients and society. The assessment summary should be worded in a way that is intelligible for non-HTA professionals. It should contain a brief executive summary, an explicit recommendation/guidance regarding the reimbursability of the medicine, the proposed reimbursement indication, and key elements for the managed entry scheme^7^ to be signed with the pharmaceutical company (see following). Whenever applicable, the guidance should refer to the reimbursement list or program in which the pharmaceutical shall be admitted, the reimbursement rate, the reference group or any other important parameter for reimbursement. The cornerstone of the assessment summary is the assessment grid, which should be accompanied by explanatory notes. The assessment summary is sent to the PRB and the pharmaceutical company.

After reviewing the assessment summary, the PRB formulates its preliminary opinion on the reimbursability of the new pharmaceutical:

- If the preliminary opinion states unconditional reimbursement, the PRB will attach to it an orientating proposal for the managed entry scheme with the pharmaceutical company (e.g., rebate or discounts per pack, patient-level caps, population-level caps, and outcome-based agreements). Next, the pharmaceutical company is notified of the PRB's preliminary opinion. In this case, the pharmaceutical company can still request to appear at a hearing where details of reimbursement can be discussed with special regard to the cost-sharing method. However, this is an option, and it should not be made mandatory. The pharmaceutical company may decide to accept the preliminary opinion in a formal confirmation after which the PRB may proceed to sanction its formal decision. The medicine cannot be dispensed with public funding/reimbursement until the managed entry scheme has been signed.
- If the preliminary opinion states conditional reimbursement or reimbursement with outcome guarantee, the pharmaceutical company is requested to participate at a PRB hearing. The pharmaceutical company is supposed to bring a detailed managed entry scheme proposal, which will be discussed alongside the assessment summary. Based on the results of the assessment summary and the PRB hearing, the PRB has the right to revise and update the assessment grid, to accept the managed entry scheme proposal, or to request a second hearing. At the latest, the finalized managed entry scheme should be signed at the second hearing. When this has been accomplished, the PRB proceeds to its formal decision.
- If the preliminary opinion states that the pharmaceutical is not admissible into public funding/reimbursement, the pharmaceutical company can request a PRB hearing to receive information about the reasons for the rejection of the reimbursement application. Right of appeal against negative HTAG recommendations should be ensured in alignment with the setup of the national health-care system/juridical system.

All PRB decisions must be made public on the Web with relevant reimbursement parameters and explanatory notes as well as the HTAG version and the final version of the assessment grid. Preferably, individual votes/opinions of PRB members should be disclosed. The decision is officially communicated to the pharmaceutical company and the health service/insurance authorities that then include the new pharmaceutical in the reimbursement list. Members of the PRB should receive competitive remuneration, and their individual performance should be subject to regular assessment.

It is crucial that decision time frames should be properly defined. We believe that a time frame of 90–120 days (after submission date) should be sufficient, with a properly designed BAS, to arrive at a well-founded pricing and reimbursement decision.^8^ Pharmaceuticals that have been found to be reimbursable should be included in the reimbursement list with no major delay. If this alternative is not viable for any reason, the reimbursement list should be updated at least twice per year on predefined dates. In a restrictive financial environment, it may also be possible that, at that time, all reimbursable new pharmaceuticals are ranked in the descending order of their total grid score, and the top-ranking ‘X’ products are admitted into reimbursement.

## Conclusions

Several MICs already use PE information in pharmaceutical pricing and reimbursement decisions, but most do it in an unstructured way. At the same time, there are trends across a lot of MICs to implement formal HTA systems (e.g., Romania, Bulgaria, Latin America), and there are also other MICs in which debate is ongoing about the reform of existing systems (e.g., Hungary).

In view of limited pharmaceutical budgets and the level of HTA resources and capabilities, pharmaceutical assessment systems in MICs should be balanced and resource conscious, while offering higher process transparency and facilitating patient access to value-added and/or cost-saving new medicines. The BAS outlined in this article may be such an approach, offering a viable compromise between decision makers’ needs and requirements and sufficient methodological coherence. The BAS approach follows a pragmatic, ‘ultralight’ stance whereby secondary assessments are sufficient, albeit primary analyses are permitted, and assessors reach back to benchmarkable prior international assessments as well as checklists in formulating their recommendations and reimbursement decision makers. The BAS system should cover a simplified economic evaluation and an assessment of value for patients and society, and it should be embedded in a transparent and linear pricing and reimbursement process.

Initially, BASs in MICs should be focused on new (on-patent) pharmaceuticals seeking reimbursement. In the later stages, systems can be extended to cover certain medical devices and surgical procedures. To support the acceptance and uptake of the system, skill development training should be organized for government officials participating in the decision-making process. Training should be differentiated based on stakeholders’ role in decision making.

In contrast to those ‘negative’ HTA systems that create time- and resource-consuming barriers to access, BASs can hopefully become examples of ‘positive’ HTA, facilitating access to value-added medicines while contributing to the sustainability of public pharmaceutical expenditures.

## Footnotes

1 In this article, for the reader's ease, we use the terms ‘reimbursement’ and ‘public funding’ interchangeably, although we acknowledge that reimbursement can also be interpreted as a subcategory of public funding.

2 Throughout the article, we use the term ‘pharmaceutical company’ to denote the entity requesting reimbursement for a pharmaceutical product. In fact, this is commonly the marketing authorization holder, its local affiliate, or an authorized distributor.

3 http://data.worldbank.org/about/country-classifications [cited 4 January 2014].

4 See e.g., http://aaz.hr/resources/radionice/29/HTA%20in%20Croatia%20Bratislava%20January%202012_ppt%20%5BCompatibility%20Mode%5D.pdf[cited 30 May 2013].

5 For an alternative approach to the basis of MCDA see, for example, Kanavos and Angelis (12) or Phillips (2012): Multicriteria decision analysis for appraising benefit-risk, http://www.mhra.gov.uk/home/groups/espolicy/documents/websiteresources/con028321.pdf [cited 4 January 2014].

6 Before and during the preparation of this article, pricing and reimbursement processes were reviewed for developed markets such as those of Australia, Canada, France, Germany, Italy, New Zealand, Spain, and Sweden, and for selected middle-income countries such as Brazil, Bulgaria, Czech Republic, Hungary, Poland, Romania, Slovakia, and Turkey, mostly on the basis of PPRI information, EMAUD educational materials, and the author's own research. This article categorically does not intend to set any of the reviewed national systems as a benchmark, and any resemblance to any national systems should be seen as a product of high-level analysis.

7 We use the term ‘managed entry scheme’ to refer to any contractual agreement between the pharmaceutical company and the payer that aims at reducing the effective net price and/or budget impact of a newly reimbursed medicine. Several classifications exist for managed entry schemes; in-practice rebate schemes, patient-level caps, population-level caps, free cycles, and outcome-based agreements can all be considered managed entry schemes.

8 This is actually shorter than the 180-day (90 +90) requirement set by the European Union's Transparency Directive. In EU countries, 180 days should be regarded as a legal maximum.

## References

[1] Swiss Network for Health Technology Assessment http://www.snhta.ch/.

[2] International Network of Agencies for Health Technology Assessment http://www.inahta.net.

[3] Garrido MV, Kristensen FB, Nielsen CP, Busse R (2008). Health technology assessment and health policy-making in Europe.

[4] EUnetHTA (2008). EUnetHTA handbook on HTA capacity building. Work package 8.

[5] Dankó D How can value be measured and valued?.

[6] Oortwijn W, Mathijssen J, Banta D (2010). The role of health technology assessment on pharmaceutical reimbursement in selected middle-income countries. Health Policy.

[7] Callahan KP, Bridges JF (2012). Using comparative effectiveness research to inform decision-making: Is there a role of economic evaluation?. J Comp Eff Res.

[8] Drummond MF, Sculpher MJ, Torrance GW, O'Brien BJ, Stoddart GL (2005). Methods for the economic evaluation of health care programmes.

[9] Cleemput I, Neyt M, Thiry N, De Laet C, Leys M (2008). Threshold values for effectiveness in health care.

[10] Donaldson C, Currie G, Mitton C (2002). Cost-effectiveness analysis in health care – contraindications. BMJ.

[11] Landa K The latest developments in P and R policy in Poland – focus on systemic efficiency. (no date).

[12] Kanavos P, Angelis A (2013). Multiple criteria decision analysis for value based assessment of new medical technologies: A conceptual framework. Working paper No. 33.

[13] O'Donnell JC, Pham SV, Pashos CL, Miller DW, Smith MD (2009). Health technology assessment in evidence-based health care reimbursement decisions around the world: An overview. Value Health.

[14] Mills A, Glied S, Smith PC (2011). Health systems in low- and middle-income countries. The Oxford handbook of health economics.

[15] ISPOR ISPOR global health care system roadmaps.

[16] Marusakova E, Bielik J Application of health technology assessment and pharmacoeconomics in the decision-making process in selected EU member states.

[17] Towse A, Buxton M (2006). Three challenges to achieving better analysis for better decisions: Generalisability, complexity and thresholds.

[18] Gulácsi L, Orlewska E, Péntek M (2012). Health economics and health technology assessment in Central and Eastern Europe: A dose of reality. Eur J Health Econ.

[19] Dankó D Assessing innovative medicines in middle-income countries.

[20] Mathauer I, Wittenbecher F (2013). Hospital payment systems based on diagnosis-related groups: Experiences in low- and middle-income countries. Bull World Health Organ.

[21] Kaló Z, Bodrogi J, Boncz I, Dózsa CS, Jóna G, Kövi R (2013). Capacity building for HTA implementation in middle-income countries: The case of Hungary. Value Health Reg Issues.

[22] Birch S, Gafni A (2006). The biggest bang for the buck or bigger bucks for the bang: The fallacy of the cost-effectiveness threshold. J Health Serv Res Policy.

[23] Le Pen C (2003). The drug budget silo mentality: The French case. Value Health.

[24] Drummond M, Barbieri M, Cook J, Glick HA, Lis J, Malik F (2009). Transferability of economic evaluations across jurisdictions: ISPOR Good Research Practices Task Force report. Value Health.

[25] Goeree R, He J, O'Reilly D, Tarride JE, Xie F, Lim M (2011). Transferability of health technology assessments and economic evaluations: A systematic review of approaches for assessment and application. Clinicoecon Outcomes Res.

[26] Kaló Z, Landa K, Dolezal T, Vokó Z (2012). Transferability of National Institute for Health and Clinical Excellence recommendations for pharmaceutical therapies in oncology to Central-Eastern European countries. Eur J Cancer Care.

